# Facilitators, best practices and barriers to integrating family planning data in Uganda’s health management information system

**DOI:** 10.1186/s12913-019-4151-9

**Published:** 2019-05-22

**Authors:** Stephen Ojiambo Wandera, Betty Kwagala, Olivia Nankinga, Patricia Ndugga, Allen Kabagenyi, Bridgit Adamou, Benjamin Kachero

**Affiliations:** 1 Department of Population Studies, School of Statistics and Planning, College of Business and Management Sciences, Makerere University, Kampala, Uganda; 2 Department of Demography & Population Studies, University of the Witwatersrand, Johannesburg, South Africa; 3 MEASURE Evaluation, University of North Carolina (UNC), Chapel Hill, USA; 4 Office of the Prime Minister, Kampala, Uganda

**Keywords:** Health information system, Uganda, Sub-Saharan Africa, DHIS, HMIS, Facilitators, Best practices, Barriers

## Abstract

**Background:**

Health management information systems (HMIS) are instrumental in addressing health delivery problems and strengthening health sectors by generating credible evidence about the health status of clients. There is paucity of studies which have explored possibilities for integrating family planning data from the public and private health sectors in Uganda’s national HMIS. This study sought to investigate the facilitators, best practices and barriers of integrating family planning data into the district and national HMIS in Uganda.

**Methods:**

We conducted a qualitative study in Kampala, Jinja, and Hoima Districts of Uganda, based on 16 key informant interviews and a multi-stakeholder dialogue workshop with 11 participants. Deductive and inductive thematic methods were used to analyze the data.

**Results:**

The technical facilitators of integrating family planning data from public and private facilities in the national and district HMIS were user-friendly software; web-based and integrated reporting; and availability of resources, including computers. Organizational facilitators included prioritizing family planning data; training staff; supportive supervision; and quarterly performance review meetings. Key behavioral facilitators were motivation and competence of staff. Collaborative networks with implementing partners were also found to be essential for improving performance and sustainability.

Significant technical barriers included limited supply of computers in lower level health facilities, complex forms, double and therefore tedious entry of data, and web-reporting challenges. Organizational barriers included limited human resources; high levels of staff attrition in private facilities; inadequate training in data collection and use; poor culture of information use; and frequent stock outs of paper-based forms. Behavioral barriers were low use of family planning data for planning purposes by district and health facility staff.

**Conclusion:**

Family planning data collection and reporting are integrated in Uganda’s district and national HMIS. Best practices included integrated reporting and performance review, among others. Limited priority and attention is given to family planning data collection at the facility and national levels. Data are not used by the health facilities that collect them. We recommend reviewing and tailoring data collection forms and ensuring their availability at health facilities. All staff involved in data reporting should be trained and regularly supervised.

**Electronic supplementary material:**

The online version of this article (10.1186/s12913-019-4151-9) contains supplementary material, which is available to authorized users.

## Background

Health management information systems (HMIS) are instrumental in addressing health delivery problems and strengthening health sectors by generating credible evidence about the health status of clients [1]. The purpose of the HMIS is to ensure consistent and systematic compilation of health data, with regular analysis and interpretation to guide key decision making and programmatic interventions [2, 3]. It is through an effective and functional HMIS that the burden of disease and utilization of services can be appropriately determined [4].

Several countries in sub-Saharan Africa (SSA) have established a national HMIS [5], with varying experiences with their implementation [6, 7]. Uganda’s HMIS is more established, formed in 1985 to collect and analyze national data on morbidity from communicable and non-communicable diseases, family planning (FP), reproductive health (RH), and immunization [2]. RH and FP data collection and reporting was initially paper-based, but is now both paper- and web-based [5, 8, 9]. The routine health data reporting system has evolved to the current platform, known as the district health information software, version 2 (DHIS 2), which began in 2011 in a few districts and was rolled out to all districts in Uganda in 2012 [8].

In Uganda, the district is considered a self-contained geography entity that presents a good opportunity for integration of multiple health programs. All public and private health facilities in Uganda are mandated to report health data to the district through the HMIS.

Family planning services in public health facilities are usually provided by the government through the national healthcare system. FP services in the private non -government sector are provided by private for-profit and private not-for-profit facilities, including faith-based organizations and non-governmental organizations (NGOs).

The Ministry of Health (MOH) manages health system in Uganda through its two national referral hospitals (namely Mulago and Butabika hospitals), regional referral hospitals, district hospitals, and health centers [3, 8].

### Problem statement

Uganda has registered improvements in some FP indicators. For instance, the modern contraceptive prevalence rate among married women increased from 8 % in 1995 to 35% in 2016. Still, contraceptive use in Uganda lags behind other countries in East Africa [10]. Sustaining the momentum of this progress requires, among other interventions, regular generation of complete and accurate FP information to facilitate appropriate programming and policy-making.

The public and private sectors independently report RH information to the national level [6, 11]. In low-income countries, the private sector is often perceived to be more efficient, accountable, and sustainable than the public sector. However, studies have shown that RH information, including FP, is not yet integrated in some countries [8, 12]. Available evidence in Uganda mainly focuses on the public sector [8].

In Uganda, FP information was captured separately from the private and public sectors until the recent adoption of a newly-integrated HMIS focusing on immunization, HIV/AIDS, and FP services [5, 8, 13, 14]. However, not all public and private health facilities are included in this new system. This has created a fragmented picture of the FP situation in Uganda – who is using FP services, how effectively are these services being provided, and where are the breaks in FP uptake – leading to a gap in the evidence base for informed decision-making.

There is paucity of studies, which have explored the facilitators, best practices and barriers to integrating FP data from the public and private health sectors into national HMIS, in sub-Saharan Africa in general and Uganda, in particular. Both facilitators and barriers can both be grouped by technical, organizational, and behavioral factors [5, 6, 8, 15–17].

### Study objectives

Therefore, the objectives of this descriptive study were to investigate the facilitators, best practices and barriers associated with the integration of FP data (i.e., public and private health sector data) into the HMIS in Uganda and develop recommendations to improve the integration of FP data.

## Data and methods

### Study design

Based on recommendations for evaluating an HMIS, we used the Performance of Routine Information System Management (PRISM) framework to guide the study [5, 6]. This was a cross-sectional, qualitative study based on key informant interviews (KIIs) and a one-day participatory multi-stakeholder dialogue (MSD) workshop. The study was conducted between 2016 and 2017.

### Sampling procedure

The study was conducted in Kampala (a national-level assessment), Jinja, and Hoima Districts. Jinja and Kampala are urban while Hoima is mostly rural. The study participants were designers of HMIS templates, users of the electronic or paper-based HMIS forms (for example medical records officers [MROs]), and FP data users. The sample size was determined based on maximum variation focusing on potential sources of data. Two districts where the DHIS 2 was implemented (i.e. Hoima and Jinja) were purposively selected [8]. A similar approach was used in Zambia [18]. From these districts, we selected three public and three private health facilities. Kampala District was included since it hosts the Ministry of Health (MOH) and therefore the national level key informants. The study recruited 27 participants, including 16 key informants and 11 MSD workshop participants. A similar approach for recruiting MSD participants has been used elsewhere [12]. Five members of the research team participated in the MSD workshop.

### Inclusion and exclusion criteria

The inclusion of study participants was based on their knowledge and experience with Uganda’s HMIS. Participants were selected from the MOH department in charge of HMIS and MROs from the MOH and public/private/NGO health facilities, FP organizations, district biostatisticians, and records officers working on the HMIS. For the MSD workshop, participant selection was also based on knowledge and experience with Uganda’s HMIS. Participants included key informants in the study and other policy or program stakeholders closely linked with HMIS.

### Data collection

Primary data collection included KIIs and the one-day MSD workshop. Participants included district officials, MOH officials, staff from public and private health facilities responsible for collection, oversight of HMIS and utilization of outcomes, and employees of donor-funded implementing partners (IPs) and stakeholders addressing FP and those with a stake in HMIS. Consent to participate in the study was obtained verbally.

### Key informant interviews

Sixteen key informants were interviewed using and KII guide (Additional file 1). The three MOH officers interviewed were from the HMIS, RH, and records departments. The interviews facilitated access and analysis of the MOH’s updated FP/RH data collection form. Interviews were aimed at assessing whether data from the two sectors are integrated for planning purposes; determining the feasibility of integrating public and private FP data; examining the consistency of data capturing in public and private sectors; and identifying facilitators and barriers of the process, lessons learned, and recommendations with respect to integration.

Three program personnel from a multi-lateral organization and NGOs working in FP were selected. The organizations were United Nations Population Fund (UNFPA), Reproductive Health Uganda, and Programme for Accessible Health Communication and Education (PACE). The researchers collected expert views on the feasibility of integrating public and private FP data.

Four biostatisticians, six MROs, and two HMIS focal persons were interviewed to establish the existence and utilization of HMIS data collection forms in the private and public facilities in the districts, the facilitators and barriers involved, and whether data from the two sectors are integrated for planning purposes (among other issues).

Six health facility-based MROs at public and private facilities that provide FP services were interviewed to assess the contents of data collection forms from the various providers for comprehensiveness and consistency, and to determine whether FP data are actually collected. This involved six health facilities: three public and three private facilities, in the two districts.

### Multi-stakeholder workshop

The one-day MSD workshop held in February 2017, was conducted to obtain an overall perspective on the content, approaches, barriers, facilitators, best practices, and recommendations for public-private FP/RH HMIS data integration (Additional file 2). The workshop involved 11 participants, who were key public and private FP stakeholders and some of the key informants. We used adult facilitation approaches such as visualization in participatory planning tools for instance brainstorming and use of cards for idea generation, clustering, prioritizing, and discussion and small group discussions [19]. The rationale for using both KIIs and the MSD workshop was to enhance triangulation of methods and validation of findings from the former.

### Data analysis

The KIIs and MSD were audio recorded, transcribed verbatim by professional transcribers, and the transcriptions were checked for accuracy by the research team. Deductive and inductive thematic data analyses were used to analyze the transcripts.

The PRISM framework, an innovative approach to designing, strengthening and evaluating routine health information systems, was used to guide the analysis. The framework supported thematic analysis by providing a systematic model for managing the data. It also guided the data coding process and presentation of results by providing a priori themes or framework for developing themes of analyzing the data [5, 6, 20].

## Results

Based on the PRISM framework analysis [5, 6], the study findings are organized based on the technical, organizational, and behavioral facilitators and barriers to integrating FP data from the private and public sectors in the national HMIS in Uganda.

### Facilitators of FP data integration in the national HMIS

#### Technical facilitators

The HMIS in Uganda is a standardized and integrated national reporting system. Standardized electronic and paper-based forms are used. Although the MOH is encouraging electronic-based data collection and reporting at all health facilities, the norm in private facilities and some lower-level and rural facilities is still to use paper-based forms due to shortages of computers, power, and internet connectivity. All health facilities (i.e., public, private for-profit, and private not-for-profit) are required to use the MOH’s standardized forms to avoid duplication of data and reduce overload of reporting to the HMIS. Records from community health workers are captured at the nearest health facility. The system requires adherence to correct procedures for compiling data and is designed for continuous cross-checking of data to eliminate errors. An example of a standardized form is HMIS Form 105 which reports monthly attendance figures for maternal and child health and FP visits, diagnoses for the outpatient department, laboratory tests, HIV and AIDS service data, stock outs of essential drugs and supplies, and financial data [21]. At a lower-level health facility, a records officer observed the following:

*Everything is incorporated within that user form. It is integrated and so FP data is catered for.*

Similarly, a national-level stakeholder observed:

*All facilities use standardized forms for reporting; the individualized and special reporting by health facilities was abolished. Thus, there is less confusion and less workload given the similar tool used in data collection at all levels.*

Most HMIS personnel at the district level had access to computers. Implementing partners, such as The AIDS Support Organization (TASO) provided computers to their partner health centers in Jinja.

The introduction of DHIS 2 by the MOH, with provisions for web-based reporting, strengthened district -based and national-level reporting. District-level HMIS personnel found the DHIS 2 software appropriate and user-friendly. Web-based reporting makes sharing health data easier among stakeholders who have user rights and can access the system. It allows IPs and donors to monitor and scrutinize the quality of data being collected. A national-level key informant made the following observation concerning HMIS:

*It is an online system that captures all the paper forms as electronic and data is sent electronically. It is a server and at the same time entry point. Biostatisticians run through the data to rectify errors. Some errors are rectified automatically but others are sent back to the facility. Web-based reporting is an innovation which does not require district HMIS officers to submit data to the ministry in hard copies, hence reducing on the transportation costs as well as minimize paper use.*

A key informant from Hoima District noted the following:

*It is one of the best data collection systems we have in the country. I have had experience overseeing Buhanguzi Health Sub-District, which has 26 facilities. The system is easily adopted by the officers, and reporting becomes easy. In particular, since 2013, there have been tremendous positive changes [in reporting].*

#### Organizational facilitators

The Government of Uganda prioritizes FP in line with its commitment to international and regional conventions (e.g., International Conference on Population and Development 1994 and FP2020). Owing to this commitment, there is substantial emphasis on improving FP service provision and consequently collecting FP data. In addition, FP is among the country’s priority areas since the contraceptive prevalence rate is monitored at the national level.

In order to ensure compliance with the HMIS, the MOH has linked submission of HMIS reports with license renewal for private health facilities. All health facilities are required to regularly submit HMIS reports to the district HMIS office. For private facilities that are not reporting consistently or fail to report, a condition stipulates that prior to the renewal of their health facility license, they must submit all missing reports to the HMIS district office. For public health facilities, when submission of HMIS reports from any one district is delayed, the records assistant receives follow-up phone calls from the district HMIS focal person asking him or her to explain the delay. At the end of the year, penalties are imposed upon districts with very poor performance.

Availability of HMIS forms in registered and licensed health facilities (i.e., private, NGO, public) facilitates the HMIS in Uganda. These forms must be submitted to the districts and, thereafter, to the MOH. FP data from community outreach programmes by private or NGO providers are captured using government health facility registers. A national-level informant noted the following concerning public facilities:

*The forms are available especially in the public health facilities. You find forms in all health facilities. Even recently, in the remotest health facility in the Karamoja Region, they had the HMIS forms.*

In addition, continuous review of the HMIS forms has been a favorable condition. Every 5 years, the MOH invites all HMIS stakeholders (e.g., UNFPA, PACE, Marie Stopes Uganda) and health unit staff (e.g., biostatisticians, records assistants) for a meeting to provide input on improving the HMIS forms.

Training designated staff in HMIS data management is a critical facilitating factor. While Hoima District reported universal training coverage, Jinja’s training coverage was reported to range from 50 to 90%. Many officers reported receiving training in HMIS data management several times (as recent as the year of data collection). Trainings address computer literacy, navigating DHIS 2, data analysis, and updates on the contents of the form. In Hoima District, all health staff attending to outpatients had been trained on HMIS data collection, including FP. A biostatistician pointed out:

*There is improvement in quality. I am not saying that [it] is very good data, but there is improvement in quality because of this regular training and introduction of this web-based reporting. I think these contribute to quality because before they would collect all the data and bring to the ministry to enter and you know what it means—and now sometimes the data entry is done at the facility.*

Supportive supervision is among the key components of the HMIS. Health facilities receive quarterly visits, mainly from district officials, and supportive visits from MOH officials. IPs, such as the World Health Organization, the United States Centers for Disease Control and Prevention, and World Vision support and participate in supervision and trainings and are usually available for consultation. Facility-level records officers receive supervisory visits from district HMIS focal persons and organizations and donors such as the United States Agency for International Development (USAID), World Health Organization, World Vision, PACE, TASO, and Infectious Diseases Institute (IDI). The MOH, through the Regional Performance Monitoring Team, conducts quarterly visits to the districts and occasionally to health facilities. Visits are followed by review meetings on how to improve service delivery. The team supports networks and linkages between IPs and health facilities.

In general, the HMIS was reported as having adequate human resources, facilities, software, and forms. In addition to government and USAID support, districts mobilize resources through collaboration with IPs. Such IPs support capacity building, training, service delivery, and commodity provision. Examples of IPs associated with FP programs in Uganda are Reproductive Health Uganda, Marie Stopes Uganda, World Vision (which also focuses on maternal, neonatal, and child health), and IDI. Monitoring and Evaluation Technology Support, a project based at the Makerere University School of Public Health that is interested in data, supports printing DHIS 2 forms for all health facilities and monitoring all data-related issues. TASO receives data from the 17 higher-level health facilities it supports. TASO staff receive free data bundles from Mobile Telecommunications Network that facilitate access to the MOH website.

#### Behavioral factors

A high level of appreciation and motivation of data users and managers is a key facilitator for enhancing HMIS performance in Uganda. IPs provided financial incentives to biostatisticians and HMIS focal persons in both Hoima and Jinja Districts. Users and managers rated the HMIS as one of the best data collection and health management systems covering different health aspects. All respondents except one were motivated to work with the system because of their interest in data analysis (if granted access), data management experience, and desire for high-quality data for planning purposes. In support of the system, a national-level officer from an NGO observed:

*No work documented is no work done. People should be encouraged to document what they have exactly done.*

Most respondents noted that health personnel were generally competent and had been cooperative in executing HMIS tasks. Relevant personnel exhibited flexibility and willingness to adapt new methods and the addenda included in the HMIS forms. Since training programs entail assessments, personnel work hard to protect their names and positions since they value their jobs. In addition, team work is promoted among the HMIS personnel, which contributes substantially to the quality of outcomes. A national-level and district-level respondent, respectively, said:

*Team work is key. Where staff work together in generation, entry and discussion of the reports has yielded quality data. In some districts, district health teams meet every end of the month to check data quality.*

*We do our work then at the end of the month, we sit on a round table. We compile, analyze, and then submit. If there are any errors, again we talk about them and you know. It is not a one man’s business.*

Collaboration or networking with other districts or actors is beneficial to the process of data management. Partnerships have made work easier and contributed to improvements in meeting reporting timelines.

With respect to consistent use of HMIS forms, national informants noted that public health facilities are more consistent in health information reporting because staff’s access to the forms is guaranteed and ensured. Unlike private facilities, public facilities receive regular supervision. An MOH informant observed that low coverage of HMIS forms at private facilities is attributed to fewer trained staff at those facilities:

*In the private sector, reporting is less than 25%. Apart from recording the name and date of review or re-attendance, nothing much is done in the private facilities. Staff need to be brought on board, to be trained and then mentored on how useful it is to capture data.*

Health facilities receive reminders from the district office when reports are due. All health facility records staff (from both private and public facilities) are expected to prepare HMIS reports for submission as stipulated in the HMIS health unit procedure manual [3]. In this manual, each health unit is expected to keep a register of all patients (Form 031) that is updated daily. From the register, tally sheets are used to populate the HMIS reporting forms. A records officer elaborated on the process as follows:

*Data are captured on a daily basis at the health facilities using the 031 form. Weekly, we report on surveillance, and that is Form 033B. We also have a monthly report and this is usually on the general condition in the facility like drugs, store medical, theatre,* etc.*, and this is HMIS 105 for outpatient and 108 for the inpatient. Quarterly, we compare three months’ information and it’s done manually. Then we have the annual report that is HMIS 107 where we generate all months and come up with one report. Finally, we have the financial year report.*

Reporting is done on a weekly basis using mobile tracing (mTrac)—a health system strengthening tool using text messaging. Data reported via text through mTrac are immediately made available within DHIS 2 for further analysis. A parallel system entails submitting hard copies of the tally sheets to the facility HMIS focal person. A provider prepares a monthly report and submits it to the district HMIS officer by the seventh of the following month for compilation and entry in DHIS 2. Monthly reports contain detailed health information from all health facilities. The district HMIS office submits all reports electronically to the MOH Resource Centre by the twenty-eighth of the following month. The MOH Resource Center disaggregates data by district, health center, and type of health facility (i.e., private or public).

Standardized and convenient data transmission procedures are used. Personnel involved in data transmission are trained in reporting procedures. Furthermore, the online reporting form makes it easy to detect mistakes. The process has been simplified with the mTrac system enabling mobile phone users to send data for weekly reports. A records assistant from Hoima reported:

*We used report on a weekly basis but right now we no longer do so because we are provided with the mTrac system. So, we can use our phones to send data. Now we send weekly reports using [the] mTrac system using Internet on our phones*.

Health facility in-charges are advised to allow records personnel to submit data directly to the district since the records personnel must receive feedback from the district on their submissions. Data in the national database can be easily retrieved and used for decision making.

Feedback at the various levels is both bottom-up and top-down. Districts provide supportive supervision to health facilities and advise staff on areas for improvement. District teams help health facilities address challenges and facilitate a process in which the worst-performing health facilities can learn from the best-performing ones. The DHIS and IPs communicate concerns about data accuracy and reporting time. Reports are graded and performance is displayed, which is a motivator for better performance. IPs also regularly share information, provide feedback, and suggest recommendations for improvement. This was particularly the case in Jinja District. The MOH provides feedback to districts on the quality of reports, data cleaning issues, reporting rates, timeliness, system issues, and performance. Coaching visits are conducted and, in such sessions, district personnel coach and obtain feedback from health facility personnel, and vice versa.

Quarterly performance reviews are conducted at the national level to provide feedback to relevant personnel. These meetings ensure that data collection and reporting is in line with HMIS data collection and reporting procedures. During performance review meetings, district HMIS focal persons and health management team members review the performance of each health facility. They then request staff from facilities that are performing well in terms of timely reporting to share with other facilities to facilitate adoption of good practices. Similarly, staff from facilities that are not performing well are requested to share their experiences, why their performance is not up to standard, and how they can be assisted. One stakeholder from Jinja District explained:

*We normally conduct performance review meetings quarterly. We look at how facilities have performed in different areas. Sometimes we spot out the best-performing facilities and the worst-performing facilities and we look at how the best ones have been managed and then we look at the worst-performing ones and what has caused them not to perform so that they tell us because of: say, “It is hard to reach,” “We are at the island so while trying to reach the place the boat got a problem and we took two weeks.” We share all that in review meetings.*

Collaboration between the MOH and IPs has been key. Although the MOH oversees HMIS implementation within the districts, it collaborates closely with IPs that support HMIS work through capacity building, training, service delivery, supervision, and commodity provision. Such IPs include USAID, the Centers for Disease Control and Prevention*,* World Health Organization, Marie Stopes Uganda, PACE, and IDI. Companies, such as the Mobile Telecommunications Network, support the HMIS by providing free data bundles to HMIS staff. This facilitates communication and results in faster access to MOH websites and improved use of DHIS 2 software. According to a national-level information officer and a records officer, respectively:

*These partnerships have “made life easy.” It is easy to address challenges, there is improvement in timelines in reporting. IPs also offer financial support.*

#### Best practices for an integrated HMIS

Based on the facilitators, we identified the following best practices for integrating public and private data into one HMIS. These best practices pertain to not only FP data, but all health data:

- **Integrating Uganda’s HMIS reporting system.** It contains all relevant health information, including FP, in one place. Consequently, there is one set of forms and no duplication of reporting in the district health information system and national HMIS.
- **Conducting routine performance reviews.** This creates opportunities where the worst performing facilities can learn from the best performing ones.
- **Enforcing data reporting compliance.** We found that conditioning renewal of licenses for private facilities on submission of required records and reports is effective for ensuring data reporting compliance.
- **Engaging stakeholders in designing and reviewing HMIS forms.** HMIS use improves when IPs and relevant partners are actively involved in developing and reviewing HMIS forms.
- **Collaborating between the government and IPs.** Systems, including the health information system, are strengthened when collaboration and networking occur between the MOH and the IPs, including the districts and civil society organizations.
- **Embracing new technology.** Demonstrating flexibility to take advantage of technology, such as using mobile phones (mTrac system) for data transfer, makes the adoption of new technology smoother. **Prioritizing staff capacity building.** This includes continuous training, and in some cases, coaching HMIS staff, supportive supervision, and provision of feedback on data reporting at various levels. **Supporting teamwork among HMIS personnel.** When team members share the workload and their data expertise, data reporting and data quality improve.

### Barriers to FP data integration in the national HMIS

#### Technical barriers

The complexity of the HMIS reporting form and procedures is challenging. Respondents noted that the reporting form is very long (too many pages and indicators), lacks adequate space for capturing data and does not have a place to indicate the sex of the FP user. Some codes used for FP data on the form are not available in Ugandan health facilities. The form does not provide explanations on meaning of the codes. Furthermore, some of the contents are not applicable to lower-level facilities. A district level informant intimated:

*The reporting form is not user-friendly. It has many pages with lots of information requirements. This makes it hectic. So, often times staff submit incomplete and inaccurate forms.*

The paper-based reporting forms (often used in rural facilities) and the online platforms have not been completely harmonized. Some of the data captured on the paper-based HMIS forms are not incorporated in the online DHIS 2. In addition, records personnel are sometimes asked to collect and capture information that is not on the paper-based form. This leads to submission of incomplete and inaccurate forms. The volume of the tool and manual is huge, which is a disincentive to staff.

Although regular revision of forms is good practice, it takes time for records personnel to become familiar with the revised forms. The implication is that if a facility uses an older version of the tool, some information is missed or not filled in.

Although two-step data entry could have value with respect to quality control, double entry of HMIS data makes the procedures strenuous. Records assistants are required to fill out hard copies of the forms that are later submitted to the district biostatistician for entry in DHIS 2. It would take less time if data were entered directly in the system and thereafter verified by the biostatistician or HMIS focal person. Some of the records officers reported that they were trained in information systems and can handle the online system.

Besides the lengthy form, some health facility personnel found the DHIS 2 software slow in generating reports. The form is designed in such a way that it does not upload when there are missing data.

Despite the availability of computers at the district level, some lower-level facilities at the sub-district level still lack computers. This presents a challenge for data entry and verification. The lack of computers increases the workload of the district biostatistician and HMIS focal person. Most facilities use traditional client registers to extract data and populate the HMIS reporting forms, which are later submitted to the district HMIS focal person. At the district level, most of the computers have old versions of computer software, lack antivirus protection, and are poorly maintained.

There are problems with Internet connectivity, which delays the transmission of data to the MOH HMIS dashboard. Similarly, inconsistent power supply at the district level delays data transmission to the MOH servers. Not all facility- or district-level staff have access to the MOH dashboard. Sometimes personnel that can access to the system and dashboard (e.g., IPs, district officers) are busy and cannot give information when it is required.

#### Organizational barriers

Staffing for the HMIS is inadequate. A senior biostatistician from the MOH reported that only five of the 15 national-level staff are available to supervise the HMIS. This is a limited number given the workload and the effort needed for effective implementation of the system. At the district level, the biostatistician is expected to capture data from all health facilities in the HMIS and perform data assessment, analysis, and validation. This heavy workload affects the timeliness, completeness, and quality of the district reports. In some health facilities, only one nurse provides FP services. Shortages in human resources result in late and under-reporting of FP data. One district-level informant said:

*I am the only person in this office who captures data from all the health facilities into the system, does data assessment, analyzes, validates, and everything else.*

Public facilities usually have fewer staff. Therefore, because of the heavy workload, staff on duty may not record FP services. Private facilities, on the other hand, may be required to report to other agencies in addition to the mandatory reporting at the district for the MOH, leading to reporting fatigue.

Constant training on the HMIS is required due to high staff turnover or attrition, especially in private facilities. A key informant from Hoima District reported that staff in private facilities frequently leave for better opportunities, resulting in recruitment of unqualified and inexperienced personnel to handle HMIS data. This creates the need to recruit and retrain new records personnel for such facilities, which is both costly and time-consuming.

When forms are revised, not everyone is trained on the changes, and this creates a barrier for data quality. Some records assistants had neither received adequate training nor refresher training. One district-level informant added:

*I have never heard of nurses and midwives going for refresher training on family planning data in the HMIS.*

Irregular training in the context of staff attrition in private facilities and revision of HMIS forms, negatively affects data quality in terms of accuracy and completeness at the district level, since responsible personnel have to learn on the job.

Several health facilities were reported to have stock outs of HMIS forms and stationery, which affects reporting. Sometimes stationery is not provided in time and districts have no budget for printing and photocopying materials for the facilities. Jinja District reportedly went without in-patient registers for a couple of years, and many facilities (both public and private) do not have FP registers. This leads to compilation of incomplete FP data since some records are made when services are provided to clients.

Registration and coding of facilities in the national HMIS by the MOH takes a long time. The district usually monitors and assesses a health facility before incorporating it in the HMIS despite commitments from IPs such as PACE. This contributes to incompleteness of data because the system is not covering all health facilities in a districts.

There is lack of support for FP data from management in some religious health facilities. For example, in some Catholic health facilities the FP section is left blank and is therefore recorded as incomplete.

#### Behavioral barriers

There is a general lack of interest in and demand for FP data on the part of IPs. Limited attention is given to FP data at the health facility level, thus affecting data quality.

Heavy workloads, especially in private health facilities, which may not have designated records staff, have the potential for FP compromising data quality. Health workers who already burdened with heavy workloads, are also responsible for record keeping. In such contexts, record keeping and documentation of FP data is often deemed a secondary priority. Hence, opportunities for checking data quality are limited.

There are challenges in collecting data on FP services provided by village health teams during community outreaches. In some cases, the services are not always recorded by health facilities. In addition, some records assistants have difficulty interpreting the FP codes that are found in the HMIS forms, leading to submission of incorrect data or information. One national-level informant reiterated:

*A case in point are entries related to condoms. So, one is required to fill in a number. However, the question is whether they are referring to packets or number of individual condoms taken by a person. So, the response expected is not clear and these data remain vague. Some records assistants focus on other indicators compared to FP, so they often have no statistics in the respective entries.*

With respect to completeness of information, informants noted that overall, reporting is around 87% for public and private facilities together. The delays are sometimes caused by lack of resources for transporting the paper-based forms to the district office, or misplacement of the forms by the relevant district staff.

Too many reports are required by the MOH, leading to heavy workloads for the HMIS focal person and district biostatisticians. IPs also have too many data demands but are given priority at the district level since they support district programs.

## Discussion

This study investigated the facilitators, best practices, and barriers associated with the integration of FP data (i.e., public and private health sector data) into the HMIS in Uganda. Qualitative data from key informants and a MSD workshop were conducted.

### Facilitators of FP integration in Uganda’s HMIS

In Uganda, the national and district HMIS are integrated with FP and RH data entered in the same system (DHIS 2). The integrated web-based reporting system addresses challenges presented by parallel systems, one of which is fragmentation of data [22]. In addition, the integration of data from both private and public hospitals has led to improved health outcomes through the monitoring of health service coverage. However, to achieve full integration, provision of adequate information technology infrastructure, ranging from first-level health facilities to national referral hospitals, should be rolled out [23].

The HMIS (DHIS 2) software was reported to be a user-friendly, and efficient web-based reporting platform. Several studies reported that user-friendly software improves data collection, transmission, and quality [6, 24–26]. On the other hand, HMIS staff at lower levels (i.e., district and health facilities) found online reporting problematic. During the peak time for submitting reports, the DHIS 2 platform tends to slow down as several users congest the web traffic. In addition, with errors and missing data, reports are inaccurate, unreliable and difficult to submit. This experience becomes challenging for the users [6, 8, 24–26].

Engaging different stakeholders in HMIS planning and design was essential success in Uganda. A study in Ghana and South Africa highlighted that consultations with different stakeholders and collaborative networks are essential for improved performance and sustainability of HMIS [22] and the success of national HMIS [24].

Our findings are consistent with the literature demonstrating that on-the-job training, with respect to Uganda’s HMIS, improves performance through timely and increased reporting of key health indicators [8]. A similar study in Uganda examining the effectiveness of the PRISM tool found it to be user-friendly, reliable, and valid. The study further demonstrated that the PRISM methodology can be used effectively by routine health management information system [5].

### Barriers to FP integration in Uganda’s HMIS

HMIS programs in SSA countries, including Uganda, face diverse challenges such as poor infrastructure, inadequate human resources, logistical issues, inadequate office equipment including computers and software, and uncoordinated collection and use of health information [8, 25, 27–30]. This often leads to incomplete and inaccurate reports, which compromise the ability to use the data to inform decisions and ultimately improve health service delivery [2, 8, 9, 31]. Our study results concurred with these findings.

This study reported long and complex HMIS forms, double entry of HMIS data and web-reporting challenges as the most critical technical challenges to FP integration in the HMIS. Not all FP data codes reflected the commodities available on the market. There was no place to indicate the sex of the FP client, which is critical for programs working to address male engagement in FP. The tedious time consuming process that entails double entry of HMIS data on the online DHIS 2 platform and paper-based versions, which demotivates health personnel is not unique to Uganda [29, 31, 32].

Our findings indicate that FP data from the HMIS are not utilized at health facilities. Priority is given to data collection and reporting to the national level. Several studies have reported a poor culture of information sharing and use related to HMIS data [18], especially at facility and district levels. This is attributed to heavy workloads, as service providers double as record officers and analysis usually takes place at a higher level, where data collectors lack access.

The importance of the various categories of data collected under FP needs to be explained to data collectors. When data quality is poor, the generated data is deemed unreliable and is therefore rarely used for planning and programmatic purposes. Hence, symbolic rather than functional use would apply [18]. A study in Tanzania reported a similar finding where HMIS data were not used at the health facility level [30]. Successful HMIS tend to be action-led where collected data are used to inform local decision making before being reported to the national level. Our findings show that use of data for planning by district and facility staff is limited [22]**.**

Although reviewing FP data collection forms is beneficial, the review should be tailored to the national context. Reporting requirements for contraceptive commodities and services that are not applicable in the national context or at various facility levels should be removed. It is important to ensure that systems collect essential rather than “nice-to-know” information [22]**.**

Our study found human resource constraints regarding HMIS implementation. The challenges are more pronounced in the private for-profit health facilities, where in some cases records officers are not hired or attrition is high [18]. Similar challenges were reported in Tanzania [30]. Although national stakeholders are generally satisfied with the tools and reporting process, facility- and district-level staff report heavy staff workloads [32]. HMIS is a laborious and prolonged reporting system [33].

## Recommendations

Based on the barriers discussed, we developed several recommendations:

- The MOH should regularly review HMIS forms so they correspond with the available FP commodities in Uganda and are aligned to the FP services provided at the health-facility level. In addition, data collection forms should provide a key for the FP codes used to collect HMIS data. During trainings, emphasis should be placed on ensuring service providers adequately understand the codes.
- The MOH should provide feedback to HMIS program designers at national and district levels to address missing FP data. This should be done to reconcile data collected on paper-based forms with data in the online forms. A review in Malawi concluded that one of the three core recommendations for improving HMIS is strengthening data collection tools [34].
- The MOH HMIS department should ensure availability of forms at the district level with follow up at lower levels to ensure that private facilities access the forms. This includes creating a budget to produce copies of forms for all public and licensed private facilities. Furthermore, districts should ensure that all health facilities (public and private) use updated HMIS forms. In addition to hard copies, soft copies of the HMIS forms in easily downloadable formats should be made available on the MOH website for public- and private-sector program personnel to download or use.
- The MOH should extend the computer-based system to the lowest-level health facilities [23]. A computerized HMIS would facilitate FP data analysis and would address issues of double entry (paper-based and online). Development partners should support the HMIS program by providing computers, internet connectivity and power backup to all eligible health facilities. Servicing of computers and replacements, where needed, is essential.
- Private health facilities should recruit required MRO staff to address the heavy workloads resulting from inadequate staffing. Approaches for motivating and incentivizing staff should be devised for better staff performance.
- The Government of Uganda should target Jinja District for initial and refresher trainings for staff. The private sector should equally benefit from the training. In addition, continuous on-the-job training of health staff in all cadres is essential to enhance performance of the HMIS, particularly when HMIS forms are revised. Pre-service trainings should adequately integrate and address HMIS concepts [30], and the HMIS should be included in training curricula [32].
- The Government of Uganda should consider reviewing the training curriculum for health professionals to integrate computer skills, as was suggested and implemented in Nigeria [35]. Persons involved in data collection at the community level (e.g., village health teams), as well as nonclinical facility-based staff, should be thoroughly trained on the data collection forms, especially for public health facilities [30].
- Trainings should aim at improving technical skills, such as information technology skills [14, 36, 37]. These efforts will build the health human resources capacity and, therefore, data quality [34]. HMIS centers of excellence, partnerships, and collaborations can be formed to deliver such trainings in Uganda [38]. There is need to train staff at district level to analyze and use data for fast response to local health issues.
- Motivation, mentorship, and supervision strategies should be devised to ensure that the records personnel, including staff at private facilities, appreciate the need for accurate, adequate, conclusive, and timely FP data in the district and national HMIS. Including the private health sector in these strategies has been recommended in Uganda [8, 14].
- More efforts should be directed towards promoting a culture of information use and sharing across health facilities, especially at the lower levels. A Ugandan study alluded to this need [5]. Regular data-use workshops have been identified as one of the best ways to promote HMIS data use [11]. HMIS personnel should be sensitized about the importance of the generated FP data.

### Strengths and limitations of the study

One of the strengths of this research is the methodological triangulation, starting with KIIs and validating the findings with an MSD workshop. This approach enabled us to verify and validate what key informants had reported as individuals, while making discussions as a team of respondents.

We explored different experiences and perceptions by interviewing a wide cross-section of HMIS stakeholders, including those from the MOH, UNFPA, NGOs, and public and private health facilities within purposively selected DHIS 2 implementing districts.

However, the key limitation of the study is that both the KIIs and MSD workshop were based on a small and purposively selected sample. As a result, the informants’ views may not be representative of all HMIS officers and users in Uganda. There is limited literature on HMIS and FP, in Uganda specifically and in SSA in general.

## Conclusions

Family planning data collection and reporting is integrated in Uganda’s district and national HMIS. The program exhibits potential for improving RH service delivery in the health system. However, limited priority and attention is given to FP data collection at both the health facility and national levels. Even when the data have been collected, they are not utilized by the relevant health facilities. Similarly, HMIS web reporting seems to be a user-friendly system, as reported by national-level stakeholders. Stakeholders at the national level, and even district officers, were impressed by Uganda’s HMIS. However, the users of the system at lower levels, such as MROs, reported experiencing a diversity of technical challenges.

Reviewing and strengthening HMIS data collection forms to reflect contextual realities is essential. The Government of Uganda should ensure availability of HMIS forms at health facilities and that all staff involved in HMIS data reporting are trained to enable them to ably support the units and track FP data inclusion. In addition, it is important to encourage and motivate HMIS staff and health facility in-charges to take interest in the FP data. The study provides important preliminary insights into factors shaping Uganda’s HMIS FP data integration process.

## Additional files

Additional file 1: Key Informant Interview (KII) Guide. (DOCX 15 kb)

Additional file 2: Multi-stakeholder (MSD) Workshop Guide. (DOCX 15 kb)

## Abbreviations

DHIS 2: District health information software, version 2
FP: Family planning
HMIS: Health management information systems
IDI: Infectious Diseases Institute
IPs: Implementing partners
KIIs: Key informant interviews
MOH: The Ministry of Health
MROs: Medical records officers
MSD: Multi-stakeholder dialogue
NGOs: Non-governmental organizations
PACE: Programme for Accessible Health Communication and Education
PRISM: Performance of Routine Information System Management
RH: Reproductive health
SSA: Several countries in sub-Saharan Africa
TASO: The AIDS Support Organization
UNFPA: United Nations Population Fund
USAID: United States Agency for International Development
