# Supplementary material for: Facilitators, best practices and barriers to integrating family planning data in Uganda’s health management information system
Source: BMC Health Serv Res. 2019 May 22;19:327. doi: 10.1186/s12913-019-4151-9 (PMC6532212; doi:10.1186/s12913-019-4151-9)
Supplement: Supplementary file 1 — Key Informant Interview (KII) Guide. (DOCX 15 kb) [file 12913_2019_4151_MOESM1_ESM.docx]

## Additional file 1: Key Informant Interview (KII) Guide

**Experiences with HMIS**

1. What is your experience with or observations about HMIS?
2. What is your experience with or observations about FP information within the HMIS?
3. What is the coverage of HMIS tools at public health facilities?
   1. What about private health facilities?
4. How consistently are the HMIS tools used in health facilities? *(Probe for public versus private.)*
5. Comment on the completeness of information generated from the tools. (*Probe for public versus private.*)
   1. Comment on the completeness of FP information generated from the tools. *(Probe for public versus private).*
6. Compared to other themes within the HMIS tool, what attention is given to FP? How does this affect:
   1. Information collection?
   2. Integration?
   3. Analysis and reporting?
7. What proportion of designated staff has been trained in HMIS data management?
   1. How does this affect the outcome?
8. What proportion of the designated staff exists at facility, district, and national levels?
   1. How does this affect the quality of data generated?

**Integration experiences and challenges**

1. How are the **data** from public and private health facilities synthesized and integrated for reporting at district and national levels? [*Request for samples of reports that have synthesized and integrated FP information.]*
   1. How are **FP data** from public and private health facilities synthesized and integrated for reporting at district and national levels?
2. What challenges do you experience in **capturing** the FP information? *(Probe for public versus private facilities.)*
3. What challenges do you experience in **integrating** FP data from public versus private facilities?
4. What challenges do you experience in **reporting** FP data from public versus private facilities?

**Approaches for integration**

1. What are the **best approaches** for integrating FP data from public and private health facilities?
2. Provide examples where integration of public and private FP data has successfully taken place at the:
   1. Facility level
   2. District level
   3. National level
3. In your view, what **facilitated the integration** of public and private FP data?
4. What can **facilitate the integration of FP data** from public and private health facilities?
   1. How should this be done?
   2. Who should take responsibility (for personnel, equipment, finances if necessary)?
   3. How feasible are these approaches?
