# Supplementary material for: Facilitators, best practices and barriers to integrating family planning data in Uganda’s health management information system
Source: BMC Health Serv Res. 2019 May 22;19:327. doi: 10.1186/s12913-019-4151-9 (PMC6532212; doi:10.1186/s12913-019-4151-9)
Supplement: Supplementary file 2 — Multi-stakeholder (MSD) Workshop Guide. (DOCX 15 kb) [file 12913_2019_4151_MOESM2_ESM.docx]

## Additional file 2: Multi-stakeholder (MSD) Workshop Guide

1. What **a**re the best practices for the integration of family planning data in the HMIS in Uganda?
2. What are the facilitators of family planning data generation and integration in the HMIS in Uganda?
3. What are the barriers to generation and utilization of family planning data in the HMIS?
4. What are the recommendations for family planning data generation and integration in the HMIS
